# Supplementary material for: Histone Demethylase KDM5C Drives Prostate Cancer Progression by Promoting EMT
Source: Cancers (Basel). 2022 Apr 8;14(8):1894. doi: 10.3390/cancers14081894 (PMC9032772; doi:10.3390/cancers14081894)
Supplement: Supplementary file 1 [file cancers-14-01894-s001.zip › cancers-1663458-supplementary.pdf]

Supplementary Materials

Supplementary methods

Histone preparation

Histone preparation was performed using acidic extraction. Briefly, cells were washed twice with cold PBS supplemented with 5mM Sodium butyrate. Afterwards, cells were resuspended in TEB (Triton extraction buffer: PBS with 0.5% Triton X 100 and protease inhibitor cocktail cOmplete Mini from Roche Diagnostics, Mannheim, Germany) for 10 min on ice and centrifuged for 10 min at 4°C at 2000 rpm. After a washing step with TEB, the pellet was incubated overnight at 4°C in 0.2 N HCl. Purified histones were analyzed by Western blotting.

Fractionated extraction

Nuclear and cytoplasmic extraction was performed with NE-PER Nuclear and Cytoplasmic Extraction kit from Pierce (#78833, Thermo Fisher Scientific) according to manufacturers' instructions. Briefly, 5x10^6 cells were washed with PBS and cell pellet was resuspended in corresponding amounts of cytoplasmic extraction reagent I containing phosphatase and protease inhibitor cocktail from Pierce (#88669, Thermo Fisher Scientific) and appropriate volume of cytoplasmic extraction reagent II. After centrifugation for 5 min at 13.000 rpm, the cytoplasmic extract was obtained from the supernatant. Following two washing steps with PBS the pellet was resuspended in suitable amount of nuclear extraction reagent and incubated on ice for 40 min with intermediate vortexing. Finally, a centrifugation step for 15 min at 13.000 rpm provided the nuclear extract. All extracts were stored at -80°C.

Phospho Kinase Array

Components of the "Proteom Profiler Array – Human Phospho-Kinase Array Kit" (R&D Systems, Inc. Minneapolis, USA) were used according to the manufacturer's protocol. Cells from one T175-flasks were pelleted by centrifugation at 800 rpm for 10 min. Pellets were then lysed with 300 µl Lysis Buffer 6 supplemented with 10 µg/ml Aprotinin, 10 µg/ml Leupeptin, and 10 µg/ml Pepstatin. Lysates were rocked gently at 4°C for 30 min and centrifugated at 14.000 x g for 5 min. The protein concentration was measured with the Pierce™ BCA Protein Assay Kit (Thermo Fisher Scientific). Proteins were stored at -80°C or used directly.

During this protocol, membranes were always incubated and washed on a rocking platform shaker. Membrane parts A and B were blocked with 1 ml of Array Buffer 1 for 1 h. 800 µg protein was used per array set and diluted with Array Buffer 1 to a final volume of 2 ml. Membrane parts were incubated overnight with 1 ml lysates each at 4°C. Membranes were washed three times with 1X Wash Buffer for 10 min and then incubated for 2 h with the Detection Antibody Cocktail A or B and 1X Array Buffer 2/3. Membranes were washed three times with 1X Wash Buffer and incubated with the Streptavidin-HRP and 1X Array Buffer 2/3 for 30 min. Membranes were washed three times with 1X Wash Buffer for 10 min. Membranes were developed using Pierce™ ECL Western Blotting (Thermo Fisher Scientific). Signals were analyzed with the Image Studio™ Light Software (Li-cor, Inc., Nebraska, USA).

RNA extraction from FFPE material for qRT-PCR

RNA was extracted from paraffin-embedded xenograft tumors using the Maxwell RSC RNA FFPE Kit (Promega, Fitchburg, Wisconsin, USA) together with the Maxwell RSC instrument (Promega), according to the manufacturer's protocol. Quantification was done with the Qubit RNA High Sensitivity (HS) Assay Kit (Thermo Fisher Scientific, Waltham, Massachusetts, USA) and measured with the Qubit fluorimeter (Thermo Fisher Scientific), according to the manufacturer's protocol.

Table S1. qPCR primers used in this publication.

Gene	Forward Primer	Reverse Primer
HPRT	5′-CAGACTTTGCTTTCCTTGGTCA	5′-ACTTCGTGGGGTCCTTTTCA
TBP	5′-AGTTCTGGGATTGTACCGCA	5′-TCCTCATGATTACCGCAGCA
KDM5C	5′-GCCAACCTTGTGCAGTGTAA	5′-TTGAACTTGGAAGGCTGCAC
CDH1	5′-AAGGGGTCTGTCATGGAAGG	5′-GGTGTTCACATCATCGTCCG
CDH2	5′-CCATCATTGCCATCCTGCTC	5′-GTTTGGCCTGGCGTTCTTTA
SNAI2	5′-TGGTTGCTTCAAGGACACAT	5′-GTTGCAGTGAGGGCAAGAA
TWIST1	5′-CCGGAGACCTAGATGTCATTGT	5′-CCCACGCCCTGTTTCTTTGA
TCF4	5′-CTTCCTCCAAACCAGCAACC	5′-CCCAACATTCCTGCATAGCC
ZEB1	5′-CAGGGAGGAGCAGTGAAAGA	5′-ACATCCTGCTTCATCTGCCT
ZEB2	5′-GACACTCTTGGCGAGGTTTT	5′-AGGCTCGATCTGCGAAGTCT
DNMT1	5′-ACCTGGCTAAAGTCAAATCC	5′-AATCACTTCCCGGTTGTAAG
EZH2	5′-CGCTTTTCTGTAGGCGATGT	5′-TGGGTGTTGCATGAAAAGAA
WNT3A	5′-GCCCCACTCGGATACTTCTT	5′-AGGAATACTGTGGCCCAACA
WNT5A	5′-ATGGCTGGAAGTGCAATGTC	5′-ACCTAGCGACCACCAAGAAT
WNT7A	5′-CTTCGGGAAGGAGCTCAAA	5′-GCAATGATGGCGTAGGTGA
WNT11	5′-CCTGTGAAGGACTCGGAACT	5′-CTTGTTGCACTGCCTGTCTT
FZD9	5′-TGGGCAGTAGTTTCCTCCTG	5′-GAAGACCCCGATCTTGACCA
AXIN2	5′-TGGCTATGTCTTTGCACCAG	5′-TTCCATCTACACTGCTGTCCG
NOTCH1	5′-CAGCCTCAACATCCCCTACA	5′-AGAACAGAAGCACAAAGGCG
JAG1	5′-GAATGGCAACAAAACTTGCAT	5′-AGCCTTGTCGGCAAATAGC
STAT6	5′-CGCAGTTCAACAAGGAGATCC	5′-TCCAGGACACCATCAAACCA
SHH	5′-ATGAAGAAAACACCGGAGCG	5′-AGTTTCACTCCTGGCCACTG
DHH	5′-ATGACCGAGCGTTGTAAGGA	5′-GGCCTTCGTAGTGGAGTGAA
GLI1	5′-CAGGGAGGAAAGCAGACTGA	5′-ACTGCTGCAGGATGACTGG
GLI2	5′-CCACGCTCTCCATGATCTCT	5′-CCAAACAGTCCCCTCTCCTT
GLI3	5′-GGACCTCAGCAACACTACCT	5′-CTGCTGCATGAAGACTGACC
HHIP	5′-GCTCGCAACGTCTCTTCATT	5′-GTCCTCTTTCATCTCCTCCCTT
BMP6	5′-AGAAGAAGGCTGGCTGGAAT	5′-GACTCCATCCCTTGTCACCA
BMP7	5′-GGGAACGCTTCGACAATGAG	5′-AAGAGATCCGATTCCCTGCC
TGFB1	5′-GCTGTACATTGACTTCCGCA	5′-GCCAGGACCTTGCTGTACT
SMAD1	5′-GCTTACCTGCCTCCTGAAGA	5′-CGCCTGAACATCTCCTCTGT
SMAD2	5′-CATCACAGCCCTCACTCACT	5′-CGCACTCCTCTTCCTATATGC
SMAD4	5′-ACTGGAAGTAGGACTGCACC	5′-TGGAAATGGGAGGCTGGAAT
SMAD7	5′-AGCCGACTCTGCGAACTAGA	5′-ATTCGTTCCCCCTGTTTCA

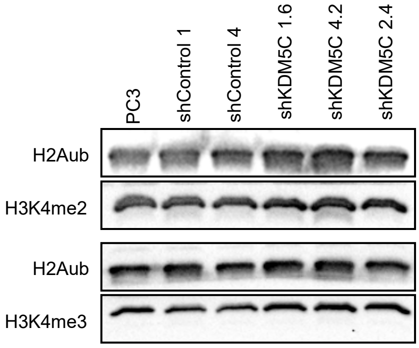

Figure S1. Western blot of the expression of the global histone methylation in histone extracts from PC3 cells and the shRNA-transduced cell clones. Western blot shows the expression of the dimethylated lysine 4 of the histone 3 (H3K4me2) and also the trimethylated type (H3K4me3) in GFP control and KDM5C knockdown clones. Ubiquityl-histone H2A (H2Aub) serves as loading control.

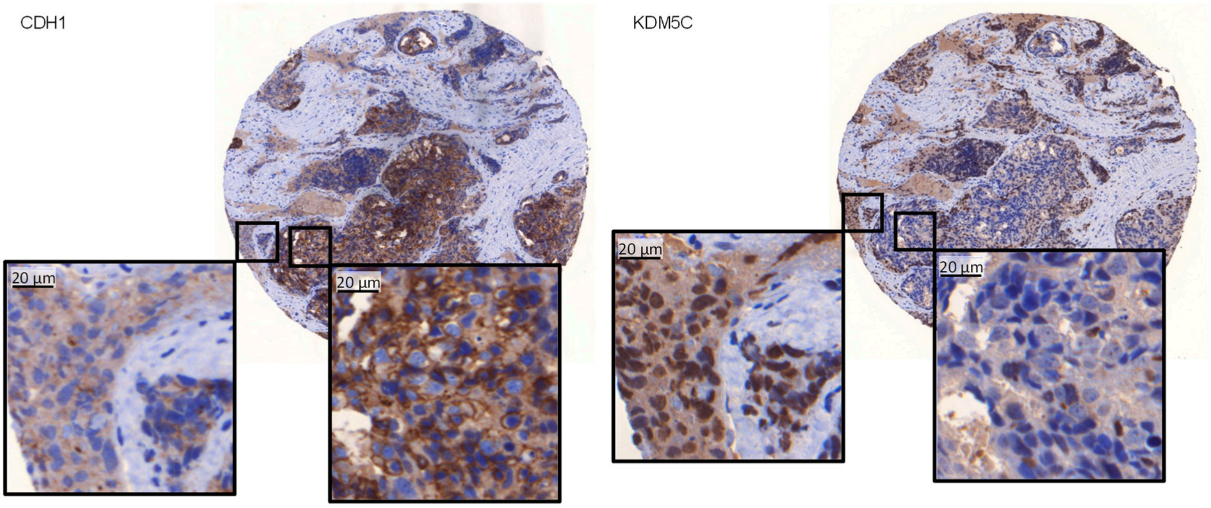

Figure S2. Negative correlation between KDM5C and E-cadherin expression in metastatic PCa patients.

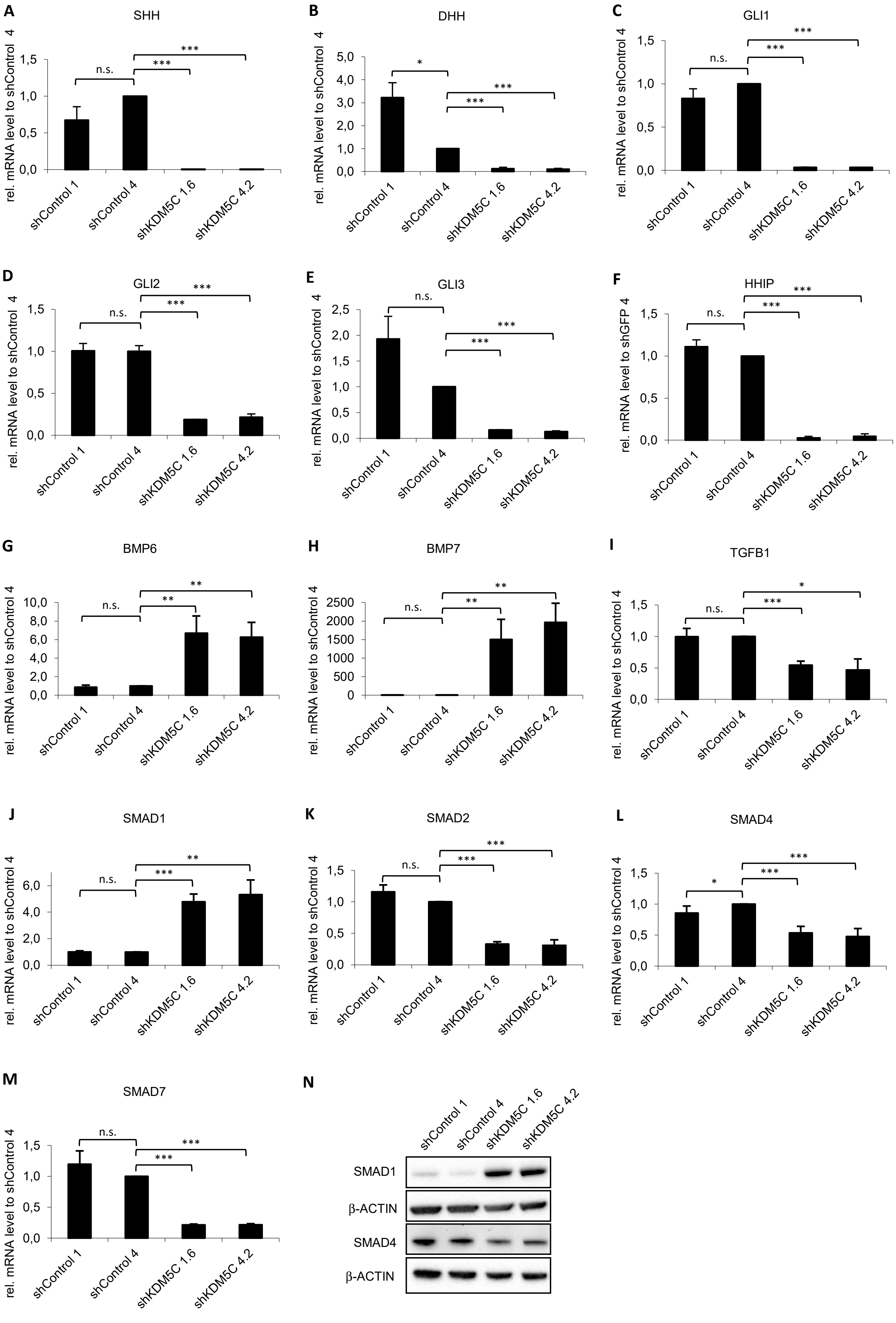

Figure S3. Expression of ligand and mediator of the Hedgehog and TGFβ signaling pathways associated with EMT progression. qRT-PCR analysis of mRNA expression in GFP control as well as KDM5C knockdown clones. Important ligands (A. SHH, B. DHH) and mediators (C. GLI1, D. GLI2,

E. GLI3, F. HIPP) for the Hedgehog signaling were examined. For the TGFβ signaling important ligands (A. BMP6, B. BMP7, C. TGFB1) and mediators (D. SMAD1, E. SMAD2, F. SMAD4, G. SMAD7) were also examined. mRNA expression was normalized to the nuclear housekeeper TBP and in relation to the shControl 4 control clone. Error bars represent standard error of the mean from three independent experiments. H. Representative western blot of the expression of SMAD1 and SMAD4 in the shRNA-transduced cell clones. β-ACTIN serves as loading control. KDM5C serves as a control of the knockdown of KDM5C in shKDM5C 1.6 and shKDM5C4.2.

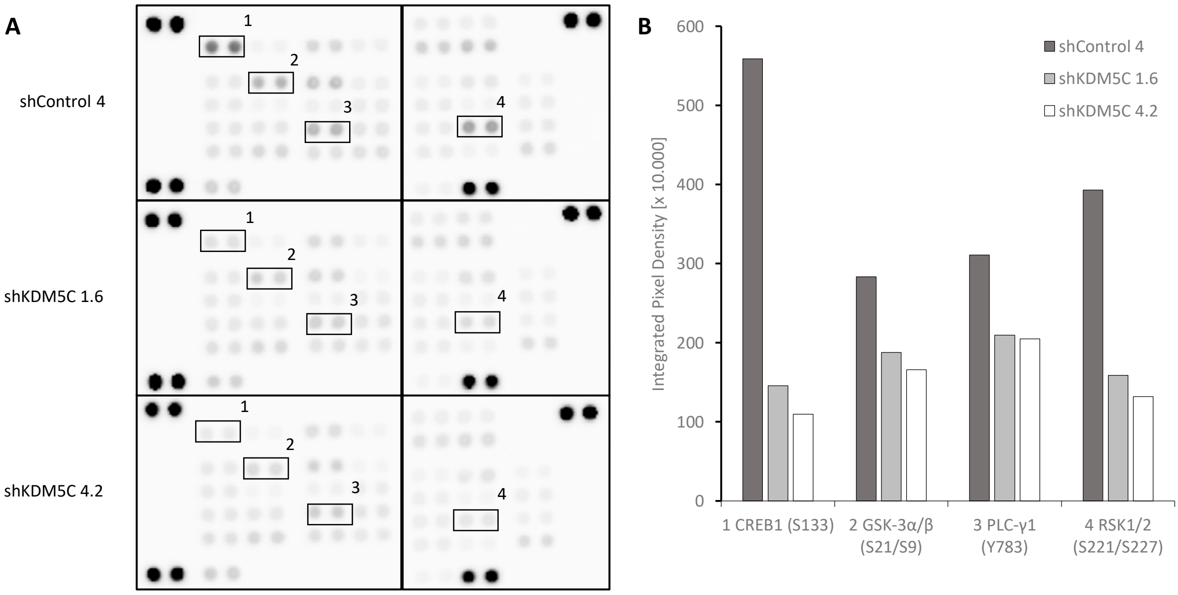

Figure S4. Differential phosphokinase-array profiles of different kinases in GFP control 4 as well as KDM5C knockdown clones. A: Images of phospho-kinase array experiments. Knockdown of KDM5C leads to reduced phosphorylation of CREB (S133, Box 1), GSK-3α/β (S21/S9, Box 2), PLC-γ1 (Y783, Box 3), and RSK1/2 (S221/S227, Box 4). B: Semi-quantitative analysis of the phosphokinase-array experiment.

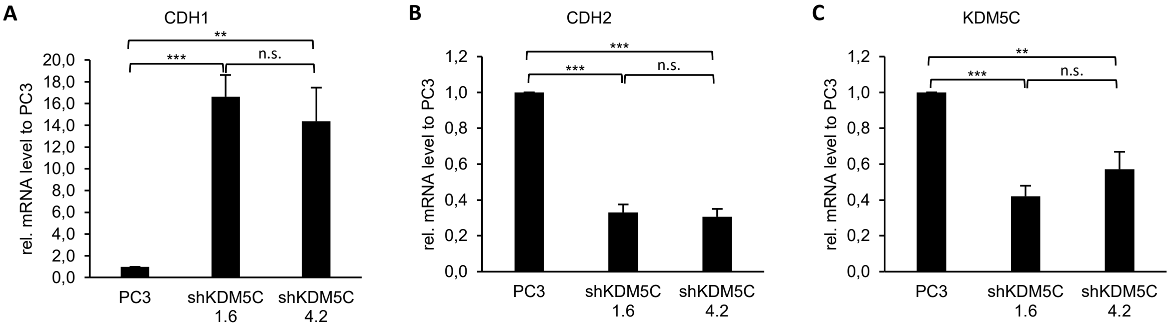

Figure S5. Expression of CDH1, CDH2 and KDM5C in mouse xenografts after termination of the experiment. Isolation of RNA from FFPE and qRT-PCR analysis mRNA expression in PC3 cells as well as KDM5C knockdown clones. mRNA expression was normalized to the housekeeper TBP and in relation to PC3. Error bars represent standard error of the mean from three independent experiments.

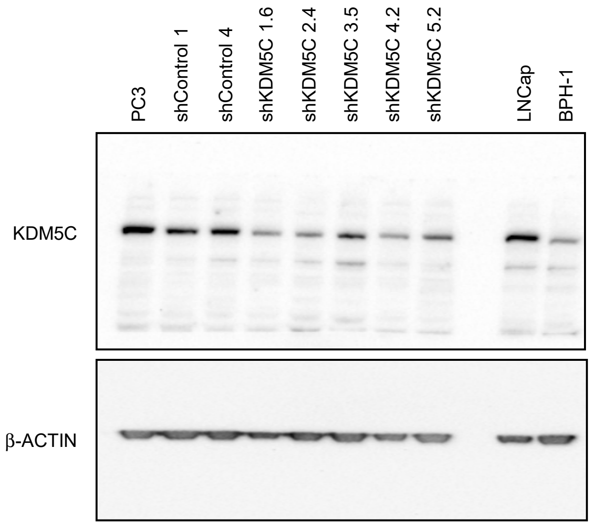

Figure S6. Uncropped western blot from figure 2A.

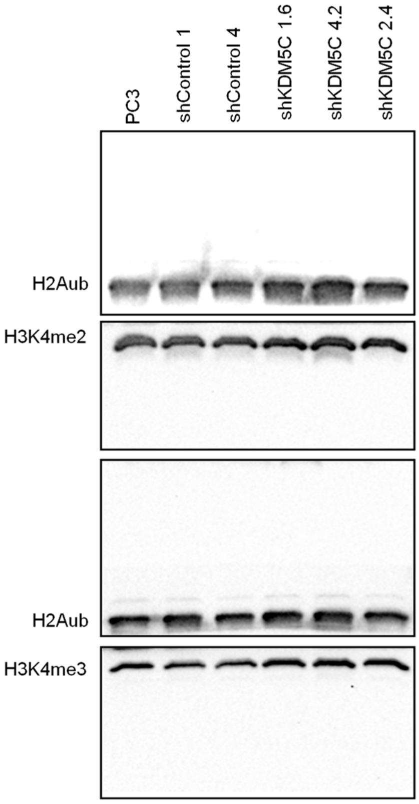

Figure S7. Uncropped western blot from figure S1.

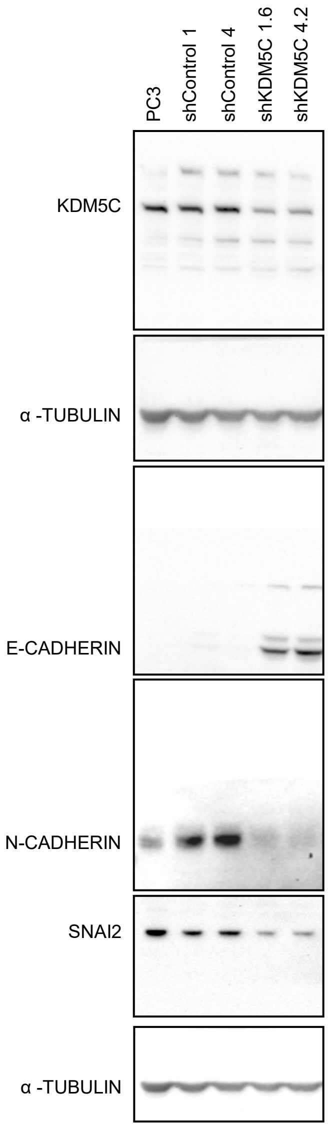

Figure S8. Uncropped western blot from figure 3D.

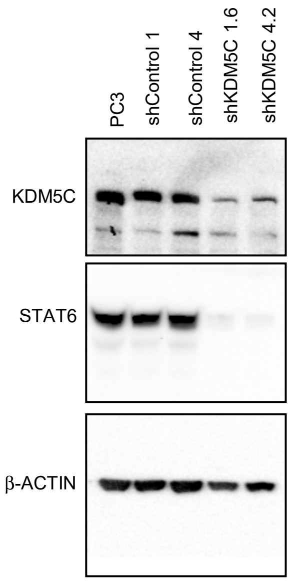

Figure S9. Uncropped western blot from figure 5J.

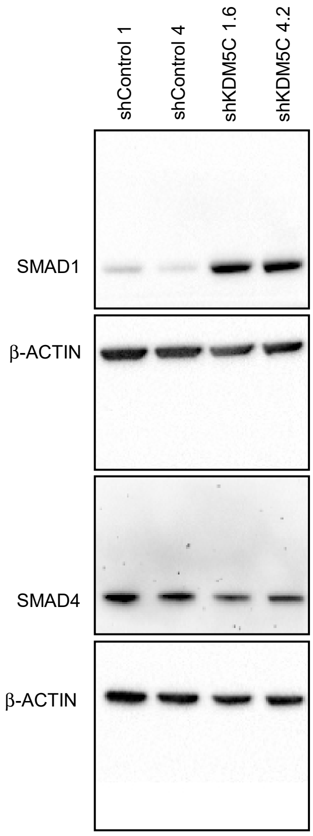

Figure S10. Uncropped western blot from figure S3N .
